# A Survey on the Knowledge, Attitude, and Practice of Students at Jazan University Regarding Calorie Menu Labeling in Restaurants and a Literature Review

**DOI:** 10.7759/cureus.61824

**Published:** 2024-06-06

**Authors:** Abdulaziz A Arishi, Nawaf Bakri, Abdulaziz Kariri, Naif Mahzara, Faisal Mahzari, Faisal Zaybi, Abdullah Alatiyyah, Abdulrahman Hadadi, Esaam Moafa, Hafiz I Al-Musawa, Hassan N Mashbari, Ibrahim A Hakami, Abdulaziz Alhazmi

**Affiliations:** 1 Department of General Surgery, Faculty of Medicine, Jazan University, Jazan, SAU; 2 Faculty of Medicine, Jazan University, Jazan, SAU; 3 Department of Microbiology and Parasitology, Faculty of Medicine, Jazan University, Jazan, SAU

**Keywords:** public health, saudi arabian, dietary decisions, body mass index (bmi), jazan university, calorie labeling

## Abstract

Background: The global rise in obesity and related health complications has cast a spotlight on the urgent need for initiatives that promote informed dietary decisions. This cross-sectional study investigates the knowledge, attitudes, and practices of university students at Jazan University, Saudi Arabia, regarding menu calorie labeling. The study examines how these variables may affect dietary decisions, body mass index (BMI), and support for proposed legislative measures requiring calorie disclosure on restaurant menus.

Methods: The study included 581 Saudi university students who were 18 years of age or older as a convenience sample. A three-part questionnaire that asked about demographics, anthropometric measurements, and attitudes and behaviors related to calorie counting was completed by the participants. Using the Statistical Product and Service Solutions (SPSS, version 25.0; IBM SPSS Statistics for Windows, Armonk, NY) program, chi-square, t-tests, and ANOVA tests were used to evaluate the data. Both informed consent and ethical approval were obtained.

Results: The study finds that, even while more than half of the participants knew their recommended daily calorie intake and exhibited curiosity about calorie information on menus, this knowledge did not always result in healthy eating habits. Participants' opinions and behaviors regarding calorie labeling were significantly correlated with their BMI levels, indicating the importance of education in promoting nutritional awareness and healthy eating habits. New calorie labeling regulations received higher approval from people who regularly ate out.

Conclusion: This study emphasizes the necessity of comprehensive nutritional education initiatives to raise calorie knowledge and encourage Saudi Arabian university students to make healthier eating choices. It also emphasizes the possible effects of legislative measures requiring calorie information on menus, particularly among regular diners. However, while evaluating the results, it is important to take into account the study's limitations, including self-reported data and convenience sample. To support menu calorie labeling legislation and inform targeted public health interventions for university students' eating behaviors, more research that takes cultural quirks and regional settings into account is necessary.

## Introduction

The objective of including nutritional information on restaurant menus is to help customers choose foods that are better for their health. Menu calorie labeling has been widely adopted during the past few years, particularly at fast-food restaurants, to encourage consumers to choose lower-calorie meal selections. Consuming foods high in calories has been connected to the emergence of obesity, type 2 diabetes, and cardiovascular illnesses [1,2].

An ongoing health issue in the Gulf countries is considerably exacerbated by overeating and choosing bad diets. According to recent studies looking at the prevalence of obesity, almost 25% of individuals in these nations - 28.52% of women and 15.5% of men, aged 20 and over - are obese [3]. Around 20% of the world's population is obese, which has epidemic proportions [4,5]. According to the World Health Organization's BMI guidelines, seven out of 10 people in Saudi Arabia are classified as overweight or obese, contributing to the country's growing obesity epidemic [6,7]. This global obesity pandemic places a heavy cost on both individual health and healthcare systems since it is directly linked to serious and preventable chronic illnesses, such as type 2 diabetes, hypertension, dyslipidemia, and coronary artery disease [5]. These non-communicable illnesses are currently being treated and prevented with an emphasis on psychological and social aspects, including nutritional control and behavioral modification.

To promote healthier dietary choices and address the growing problems of obesity and chronic illnesses, menu calorie labeling rules have been implemented in several nations, including the United States and Saudi Arabia [8,9]. While the Saudi Food and Drug Authority (SFDA) in Saudi Arabia introduced this requirement in 2017 as part of their 2030 vision for improving consumer eating habits, the US Food and Drug Administration (FDA) enforced menu calorie labeling for large food chain establishments in compliance with the Affordable Care Act in 2018 [10]. As a measure to increase consumer knowledge and maybe reduce the prevalence of obesity, this regulation requires restaurants to show calorie information for menu items. Restaurants have redesigned their menus to offer healthier options as a result of research showing that it is a cost-effective strategy with advantages for healthcare and society [11]. Similar rules have been enacted in Europe, where food places must now display calorie and allergy information [12].

The effects of menu calorie labeling regulations on consumer energy consumption have been the subject of several research studies [13-15]. According to a study conducted by Brissette et al. [16], people are more likely to choose lower-calorie options in fast-food restaurants when calorie information and awareness are promoted. Calorie labeling is viewed as a cutting-edge tactic that has the potential to transform the food landscape and raise consumer awareness of calorie intake, perhaps helping to reduce the expenses associated with obesity [17]. Energy-dense food intake at restaurants is often linked to greater amounts of sugar, cholesterol, and saturated fat, creating an obesogenic environment and increasing the risk of lifestyle illnesses, including diabetes and cardiovascular disease [18]. These studies have produced somewhat contradictory findings, with some pointing to somewhat good impacts of calorie labeling in cafeteria settings and others indicating insignificant or limited effects, particularly in fast food restaurants [19,20]. Consumers face many challenges that prevent them from using calorie labeling, including issues with cost, time management, difficulty understanding calorie information, and the impact of factors such as hunger, individual preferences, and ingrained ordering patterns [21,22].

Despite the acknowledged importance and necessity of such a study, Saudi Arabia has not received much attention in relation to this problem. Furthermore, it is critical to assess the effectiveness of this strategy, particularly as consumers become more aware of the connections between their food decisions and health consequences. It is crucial to raise people's knowledge of the nutritional implications of calorie labeling. Recognizing and comprehending these obstacles is also essential for making suggestions that are applicable and useful to people in charge of food labeling. Thus, using a knowledge-attitude-practice method, our study aims to evaluate the awareness, knowledge, and behavior of students at Jazan University about menu calorie labeling in restaurants. Our goal also includes figuring out what prevents Saudi Arabian customers from using menus that list calories.

## Materials and methods

Study design, sample size, and setting

The research was structured as a descriptive cross-sectional study. An examination of the knowledge, attitudes, and habits of 581 students at Jazan University in Saudi Arabia regarding menu calorie labeling was done using a convenience sample. Participants in this study are adult students, above the age of 18, who were enrolled at Jazan University. With "z" standing for the critical value from the standard normal distribution for the selected confidence level, "P" standing for the estimated proportion of the population with the characteristic of interest (which was 0.38), "1 - P" standing for the estimated proportion without the characteristic of interest, and "d" standing for the allowable margin of error (which was 0.05), the sample size was calculated. The right sample size was determined in this instance to guarantee a 5% margin of error, and a 95% confidence interval, and to fairly represent the existing student body at Jazan University (581).

Instrument

We developed a three-part questionnaire based on our research objectives, drawing insights from previous studies on calorie tracking in dietary habits [11,23]. The first section collected demographic and academic information from 581 participants, including age, gender, nationality, college affiliation, academic level, marital status, and location. In the second segment, we gathered anthropometric data, such as height and weight, as well as information on physical activities such as running, swimming, and playing football. The third part delved into university students' attitudes and behaviors concerning calorie monitoring in their diets. We investigated their beliefs regarding the importance of counting calories in their diets, whether they engaged in calorie counting, their familiarity with recommended daily calorie intake for their bodies, and their perspectives on the significance of calorie labeling on restaurant menus.

Data collection procedure

We chose to gather data via an anonymous, web-based, self-administered questionnaire due to the sensitivity of some parts of the questionnaire. The student body at Jazan University was carefully provided with a copy of this questionnaire. By delivering it directly to a few chosen individuals through email or messaging, we helped spread it. The questionnaire was completed by students on their preferred computing devices, such as laptops and mobile phones. With respect to our university student participants' accessibility and choices, this method offered a smooth and private data-gathering procedure.

Pretesting

A total of 25 students participated in a pilot study aimed at testing the validity of the data and examining the phrasing of the questionnaire. This procedure was designed to enhance the understandability and comprehensiveness of the questionnaire, thereby reducing potential biases and ensuring its validity. Participants involved in the pilot study were excluded from the main study to maintain the integrity of the data. Based on the results of the pilot research, necessary revisions were implemented. After these revisions, all variables demonstrated a validity index greater than 0.7, which was statistically significant (P<0.05).

Data and statistical analysis

The Statistical Package for Social Sciences (SPSS, version 25.0; IBM SPSS Statistics for Windows, Armonk, NY) program was used to enter and analyze all of the data. The representation of categorical variables, such as gender, age, and nationality, used numerical values and percentages. Students' BMI categories were established by dividing weight by height square to get BMI. The chi-square test was used to determine if categorical variables were statistically significant. T-tests and ANOVA tests were also carried out to assess the mean knowledge and awareness levels among the pupils. A p-value of less than 0.05 was used as the threshold for statistical significance in the studies.

Ethics approval and consent to participate

To guarantee adherence to ethical norms, ethical permission is requested from the Jazan University Research Ethical Committee (REC-44/4/370). In order to respect the participants' autonomy, the questionnaire maintained respondent anonymity and contained a section at the outset asking for informed consent. Participants will be given an informed consent form before beginning the study, which specifically outlines its goals, methods, and potential effects. Participants were asked to declare their willingness to take part in the research in order to give their voluntary permission. This procedure for obtaining permission demonstrated the dedication to maintaining participant autonomy throughout the study.

## Results

**Figure 1:**
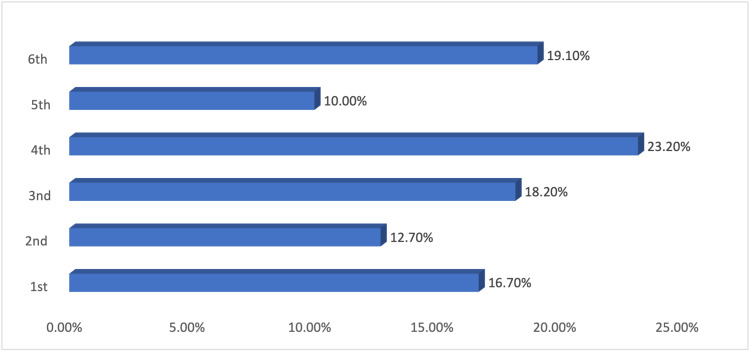
Education level in college.

The demographic breakdown of the 581 participants is detailed in Table 1, outlining factors such as gender, age, nationality, educational background and level (Figure 1), residence, marital status, and BMI. The majority of the respondents were Saudi, single, and lived in villages. Around half had a healthy BMI, and the rest were evenly distributed across various BMI categories.

**Table 1:** Demographic characteristics and BMI distribution of the study participants. The data have been represented as frequency (N) and percentage (%).

Variables	Frequency	Percent
Age (years)		
18-20	193	33.2
21-23	295	50.8
24 and more	93	16
Gender		
Male	354	60.9
Female	227	39.1
Nationality		
Saudi	578	99.5
Non-Saudi	3	0.5
College		
Medical collage	285	49.1
Non-medical collage	296	50.9
Level		
1st Year	97	16.7
2nd Year	74	12.7
3rd Year	106	18.2
4th Year	135	23.2
5th Year	58	10
6th Year	111	19.1
Place		
City	234	40.3
Village	347	59.7
Marital status		
Single	526	90.5
Married	50	8.6
Divorced	5	0.9
Body mass index (BMI)		
Underweight	91	15.7
Healthy	314	54
Overweight	106	18.2
Obese	70	12

Table 2 analyzes the participants' exercise habits, calorie knowledge, and opinions on calorie labeling in restaurant menus. While walking was the most popular form of exercise, about a third did not engage in any sports. Awareness of daily calorie intake was split, with 44.8% being knowledgeable. Although most recognized the presence of calorie labels on menus, only 35.1% admitted that it influenced their dietary choices. The majority agreed that menus should include calorie counts.

**Table 2:** Assessment of knowledge, attitudes, and practices of students regarding menu calorie labeling. The data have been represented as frequency (N) and percentage (%). T-tests and ANOVA tests were also carried out to assess the mean knowledge and awareness levels among the students.

Variables	Frequency	Percent
How often do you exercise during the week?		
I don't do sports	188	32.3
Less than twice a week	170	29.3
Three times a week	93	16
Four times a week	48	8.3
Five times or more	82	14.1
What type of exercise do you do?		
Walking	201	50.8
Soccer	107	27
Swimming	5	1.3
Weightlifting	62	15.7
Other sports	21	5.3
Do you know the recommended daily caloric intake for your body?		
Yes	260	44.8
No	321	55.2
Have you noticed that there are calories displayed on food menus?		
Yes	494	85
No	87	15
Do you use calorie information in restaurants and other products to determine your diet?		
Yes	204	35.1
No	377	64.9
When choosing a meal in a restaurant, how keen are you to note the number of calories in it?		
Always	53	26
Mostly	66	32.4
Sometimes	63	30.9
Rarely	22	10.8
Does the number of calories determine your diet		
Yes	176	30.3
No	405	69.7
In your opinion, is the calorie classification of menus important to the consumer?		
Strongly agree	275	47.3
Agree	187	32.2
Neutral	98	16.9
Disagree	12	2.1
Strongly disagree	9	1.5
How many times a week do you eat (go out) in restaurants?		
Once	181	31.2
(2-4) Times	276	47.5
(5-7) Times	80	13.8
More than 7 times	44	7.6
Do you support the new policy requiring restaurants to display calories on their menus?		
Strongly agree	411	70.7
Agree	128	22
Neutral	32	5.5
Disagree	3	0.5
Strongly disagree	7	1.2

The study revealed gender disparities in BMI profiles, with females generally having healthier BMIs than males. More females paid attention to calorie information when choosing meals, a factor that might contribute to better BMI profiles. However, there was no significant gender difference in the perceived importance of calorie labeling on menus, as demonstrated in Table 3.

**Table 3:** Attitudes and practices of participants based on their gender. The data have been represented as frequency (N) and percentage (%). A p-value less than 0.05 has been considered statistically significant.

Variables	Gender				P-value
	Male		Female		
	N	%	N	%	
BMI					
Underweight	48	13.6	43	18.9	0.004
Healthy	181	51.1	133	58.6	
Overweight	71	20.1	35	15.4	
Obese	54	15.2	16	7.1	
How often do you exercise during the week?					
I don’t do sports	86	24.3	102	44.9	<0.0001
Less than twice a week	108	30.5	62	27.3	
Three times a week	62	17.5	31	13.7	
Four times a week	32	9	16	7	
Five times a week	66	18.6	16	7	
What type of exercise do you do?					
Walking	103	38.3	98	77.1	<0.0001
Soccer	103	38.3	4	3.1	
Swimming	2	0.7	3	2.4	
Weightlifting	47	17.5	15	11.8	
Other sports	14	5.2	7	5.5	
Do you know the recommended daily caloric intake for your body?					
Yes	151	42.7	109	48	0.231
No	203	57.3	118	52	
Have you noticed that there are calories displayed on food menus?					
Yes	303	85.6	191	84.1	0.635
No	51	14.4	36	15.9	
Do you use calorie information in restaurants and other products to determine your diet?					
Yes	122	34.5	82	36.1	0.722
No	232	65.5	145	63.9	
When choosing a meal in a restaurant, how keen are you to note the amount of calories in it?					
Always	25	20.5	28	34.1	0.059
Mostly	46	37.7	20	24.4	
Sometimes	40	32.8	23	28	
Rarely	11	9	11	13.4	
Does the amount of calories determine your diet?					
Yes	110	31.1	66	29.1	0.644
No	244	68.9	161	70.9	
In your opinion, is the calorie classification of menus important to the consumer?					
Strongly agree	168	47.5	107	47.1	0.130
agree	124	35	63	27.8	
Neutral	53	15	45	19.8	
Disagree	5	1.4	7	3.1	
Strongly disagree	4	1.1	5	2.2	
How many times a week do you eat (go out) in restaurants?					
Once	73	20.6	108	47.6	<0.0001
(2-4) times	187	52.8	89	39.2	
(5-7) times	59	16.7	21	9.3	
More than 7 times	35	9.9	9	4	
Do you support the new policy requiring restaurants to display calories on their menus?					
Strongly agree	261	73.7	150	66.1	0.266
Agree	70	19.8	58	25.6	
Neutral	16	4.5	16	7	
Disagree	2	0.6	1	0.4	
Strongly disagree	5	1.4	2	0.9	

A noticeable trend was the positive correlation between regular exercise and awareness of calorie content in food. Walking enthusiasts particularly showed strong agreement with the necessity of calorie information on menus. On the contrary, those favoring weightlifting or soccer did not prioritize calorie information as much. Individuals informed about their daily caloric requirements showed a slightly stronger preference for calorie labeling, although this was not statistically significant.

Gender did not markedly influence the utilization of calorie information in dietary decisions, with a significant portion of both groups not relying on such data. The survey revealed a tendency among females to be slightly more attentive to calorie information, hinting at a potential area for further research.

Despite the non-significant gender difference in using calorie content to dictate diet, the data underscored that a substantial segment of both groups did not prioritize calorie information in their food choices. The study suggested that both males and females value calorie information on menus, albeit males showed a slightly stronger agreement on its importance.

Regarding dining out habits, a significant gender difference was found; males dined out more frequently than females. This gender-based discrepancy in dining frequencies could offer a foundation for further investigations into its impact on health-related behaviors.

Table 4 also shows a statistically significant relationship between individuals' calorie labeling habits and the participant’s educational attainment (0.007). Regarding dining out habits, a significant gender difference was found; males dined out more frequently than females. This gender-based discrepancy in dining frequencies could offer a foundation for further investigations into its impact on health-related behaviors.

**Table 4:** Association between education attainment and participants' calorie labeling habits.

Variable	Yes, n (%)		No, n (%)		P-value
1st Year	77	15.6%	20	23.0%	0.007
2nd Year	59	11.9%	15	17.2%	
3rd Year	89	18.0%	17	19.5%	
4th Year	117	23.7%	18	20.7%	
5th Year	52	10.5%	6	6.9%	
6th Year	111	19.1	111	19.1	

The results also show the presence of a positive correlation between regular exercise and awareness of calorie content in food (p=0.0001; Table 5). Walking enthusiasts particularly showed strong agreement with the necessity of calorie information on menus 148 (51.0%). On the contrary, those favoring weightlifting, swimming, and other sports did not prioritize calorie information as much: 52 (17.9%), 19 (6.6%), and three (1.0%), respectively. However, the difference was not statistically significant (p>0.05).

**Table 5:** Association between the type of exercise and the need for calorie information in the menu.

Variable	Strongly Agree	Agree	Neutral	Disagree	Strongly Disagree	P-value
Walking	148 (51.0%)	40 (50.0%)	9 (50.0%)	1 (50.0%)	3 (50.0%)	0.376
Soccer	68 (23.4%)	30 (37.5%)	7 (38.9%)	0 (0.0%)	2 (33.3%)	
Swimming	3 (1.0%)	2 (2.5%)	0 (0.0%)	0 (0.0%)	0 (0.0%)	
Weightlifting	52 (17.9%)	6 (7.5%)	2 (11.1%)	1 (1.6%)	1 (1.6%)	
Other sports	19 (6.6%)	2 (2.5%)	0 (0.0%)	0 (0.0%)	0 (0.0%)	

## Discussion

This research study investigated the knowledge, attitudes, and practices of students of the University of Jazan regarding the calorie labeling of menus in restaurants in Saudi Arabia. The attitudes, practices, and hindrances of menu labeling are identified on the basis of responses that were self-reported by the participants. The data demonstrated that nearly more than half of the participants knew their daily calorie intakes while the other half had nearly no knowledge of their daily calorie requirements. The data suggested that the latter half does not have adequate knowledge about the calorie intake. These findings were in accordance with the studies that were also conducted in Saudi Arabia and the USA [23,24].

In a similar pattern, most of the participants stated that they are keen to know the number of calories that are present in their food and notice when the calories are mentioned on the food menu. Furthermore, it has been demonstrated that most of the participants are not affected by the number of calories in their food even though they are interested in knowing the number of calories in their food. Consumers in Saudi Arabia may choose healthier foods if educational programs are implemented that are designed to increase calorie knowledge and usage. It is noteworthy that the survey participants in this study showed positive opinions and support for menu calorie labeling, highlighting its potential value. The effective execution of such a strategy depends critically on these favorable attitudes among the populace. According to Block et al., customer receptivity has a considerable impact on the efficacy of menu labeling regulations, making it an important factor to take into account when developing and implementing rules [25]. These findings suggest a potential for awareness-raising and educational initiatives to help people, regardless of gender, better comprehend and regulate their daily caloric intake. Participants in the survey can make better food choices and reach their overall health objectives by promoting nutritional knowledge [26].

Given the variations in exercise patterns, our findings highlight the necessity for focused programs to encourage physical activity across both genders. Initiatives to increase female engagement in sports and exercise may be beneficial, while male efforts may concentrate on maintaining their regular exercise schedules. Regardless of gender, the ultimate objective is to promote a healthy level of physical activity among all participants. The data reveal an interesting pattern, with almost 47% of the individuals saying they eat out two to four times each week. This is consistent with research from a previous cross-sectional study of 196 Saudi teenagers, in which almost half of the participants admitted to eating fast food at least once per week and 20% reported doing so more frequently than twice per week [27]. Furthermore, according to a different study, Saudi teenagers consume a lot of high-fat fast food. Similarly, Jeddah-based research found that Saudi citizens frequently consume junk food and fast food [28]. Between one and three times each week, both adults and teenagers consume fast food, putting them at risk for a variety of health problems [29]. These findings show how important it is to change Saudis' eating habits and encourage them to make better decisions. 

Our research demonstrates substantial correlations between the attitudes and behaviors of individuals and their BMI levels, correlating with research from India that found a significant relationship between high BMI and fast food intake [30]. This underlines the potential effects of calorie labeling in fast-food restaurants as it may influence customers to choose lower-calorie options or make calorie-aware menu selections. Interestingly, we also discovered a statistically significant relationship between individuals' calorie labeling habits and their educational attainment (0.007). This conclusion underscores that people with greater levels of education often have more positive habits when it comes to calorie labeling and is consistent with comparable findings in other research [31]. These results imply that education might be a key factor in raising calorie information knowledge and use, particularly in restaurant settings. Furthermore, our research shows that opinions regarding menu calorie labeling are significantly predicted by both BMI levels and educational attainment. These variables interact because having more knowledge may lead to making better-informed decisions, which may have an impact on BMI levels. This interaction between education, awareness, and BMI emphasizes the intricate interplay between socioeconomic variables and health-related behaviors, underscoring the necessity for multiple interventions targeted at enhancing food preferences and encouraging healthier lifestyles across a variety of communities.

Participants in our study's propensity for eating out has important ramifications for how they feel about the new rule forcing eateries to list calories on their menus. According to the statistics, 52.8% of respondents go out to eat two to four times per week, compared to 48.5% of respondents who dine out once per week. Importantly, there is a statistically significant correlation between individuals' opinions regarding the calorie labeling legislation and how frequently they eat out (p-value = 0.0001). Surprisingly, compared to those who eat out less frequently, those who eat out two to four times a week exhibit more support for the policy. This finding shows that those who dine out more frequently could place a larger value on having access to calorie information when choosing what to eat. They are probably going to see calorie labeling as a useful tool for regulating their calorie intake in the context of their eating patterns and making educated dietary decisions. As a result, this association highlights the usefulness of calorie labeling laws in meeting the requirements and preferences of those who often eat at restaurants [13]. These findings highlight the linked relationship between dietary preferences and policy opinions, illuminating how individuals' frequency of restaurant eating might affect their openness to campaigns that promote calorie information. Such information is useful for public health advocates and legislators who want to develop measures that would increase nutritional knowledge and encourage people to eat better in restaurants, especially regular diners [32].

When navigating the calorie labels on restaurant menus, university students encounter a specific set of difficulties. Even though there has not been much research on this group specifically, it is obvious that there are many different types of obstacles. First and foremost, many students do not have a thorough understanding of nutrition, which is essential for comprehending and efficiently utilizing calorie information. It can be difficult to grasp the meaning of calorie numbers and their relation to individual dietary demands without a strong background in nutrition [33]. University students frequently deal with busy academic schedules and a variety of obligations, which can lead to time limitations that make it difficult to make smart eating choices. Consideration of the calorie amount of menu items may be subordinate to the demand for quick, simple meals. Financial difficulties can also be a big obstacle because students sometimes look for inexpensive solutions that may not be calorie-conscious. Educational institutions must take into account comprehensive nutrition education and support services that provide students with the information and abilities to make better eating choices, even in the hectic environment of a university. This will help solve these difficulties [34]. While there may not be many studies specifically concentrating on university students and calorie labeling, general studies on calorie labeling hurdles and more general university student problems offer important insights into potential challenges this group may experience. In the context of calorie labeling programs, more studies could provide more focused solutions to promote healthy eating habits among university students [34].

The convenience sampling technique used in this study, which could not accurately reflect the opinions and behaviors of the general public regarding menu calorie labeling, is one of its limitations. Additionally, the study's dependence on self-reported data might introduce response bias since participants can give responses, they believe would make them look good in front of others rather than ones that accurately reflect their actual actions and viewpoints. Our capacity to establish cause and effect or monitor changes over time is constrained by the cross-sectional design. The conclusions of the study cannot be applied to other people or areas of Saudi Arabia because of the study's exclusive emphasis on a particular university. The study also did not examine any cultural or geographical variations in views and practices, which would have shed light on the usefulness of menu calorie labeling in various situations.

## Conclusions

This study sheds light on the misconceptions, perspectives, and actions of college students at Jazan University in Saudi Arabia with regard to menu calorie labeling. It emphasizes the requirement for extensive nutritional education programs designed to raise pupils' calorie knowledge. Although a sizable majority of participants expressed interest in understanding the calories shown on food menus, this awareness did not always result in improved dietary choices, indicating the existence of obstacles that need to be removed. The study also found a link between individuals' beliefs and behaviors regarding calorie labels and their BMI levels, educational backgrounds, and BMI levels. Education has a crucial role in promoting nutritional knowledge and healthy eating habits, as seen by the correlation between higher educational attainment and more beneficial practices. The study also found that those who frequent restaurants are more likely to favor the requirement for menus to list calories, underscoring the potential influence of such legislation in promoting educated dietary choices among frequent restaurant clients. It is important to recognize the study's constraints, though, including the use of convenience sampling and self-reported data, which might create biases and restrict the applicability of the findings. In order to create more focused and efficient public health initiatives for encouraging healthy eating habits among university students, future research should dive further into these processes while taking cultural variances and regional settings into account.

## Appendices

This questionnaire was used to measure awareness among the students of Jazan University regarding the amount of calories.

**Table 6:** Personal characteristics.

	Category	Choices	Choose here
Q 1	Gender	Male	
		Female	
Q 2	Age (Years)		
Q 3	Nationality	Saudi	
		Non-Saudi	
Q 4	Faculty		
Q 5	Level	First year	
		Second year	
		Third year	
		Fourth year	
		Fifth year	
		Sixth year	
Q 6	Housing	Urban	
		Rural	
Q 7	Social status	Single	
		Married	
		Divorced	
		Widower	

**Table 7:** The body mass index (BMI) anthropometric measurement is a measure of body fat based on height and weight, i.e., the ratio of weight to squared height.

	Category	Choices		Choose here
Q 8	Hight :__ cm Weight :__ kg			
Q 9	How many times do you exercise during the week: (If the answer is no, skip the next question)?	Yes		
		No		
Q 10	What kind of exercise do you do?	Walking		
		Football		
		Swimming		
		Lifting weights		
		Others		
	Category	Choices	Choose here	
Q 11	Do you know the recommended daily intake of calories for your body?	Yes		
		No		
Q 12	Have you noticed that there are calories displayed on the menus?	Yes		
		No		
Q 13	Q14- Do you use calorie information in restaurants and other products? To determine your diet?* (If your answer is no, skip the next question)?	Yes		
		No		
Q 14	When choosing a meal in a restaurant, how keen are you to note the amount of calories in it?	1] Always		
		Often		
		Sometimes		
		Rarely		
Q 15	Does the amount of calories determine your diet?	Yes		
		No		
Q 16	In your opinion, is the classification of calories in menus important to the consumer?	1- Strongly agree		
		2- I agree		
		3- Neutral		
		4- I disagree		
		5- I strongly disagree		

	Category	Choices	Choose here
Q 11	Do you know the recommended daily intake of calories for your body?	Yes	
		No	
Q 12	Have you noticed that there are calories displayed on the menus?	Yes	
		No	
Q 13	Q14- Do you use calorie information in restaurants and other products? To determine your diet?* (If your answer is no, skip the next question)?	Yes	
		No	
Q 14	When choosing a meal in a restaurant, how keen are you to note the amount of calories in it?	1] Always	
		Often	
		Sometimes	
		Rarely	
Q 15	Does the amount of calories determine your diet?	Yes	
		No	
Q 16	In your opinion, is the classification of calories in menus important to the consumer?	1- Strongly agree	
		2- I agree	
		3- Neutral	
		4- I disagree	
		5- I strongly disagree	

	Category	Choices	Choose here
Q 17	How many times a week do you eat (go out) in restaurants?	Once	
		2- (2-4) times	
		3- (5-7) times	
		4- More than 7 times	
Q 18	Do you support the new policy that requires restaurants to display calories on their menus?	1- Strongly agree	
		2- I agree	
		3- Neutral	
		4- I disagree	
		5- I strongly disagree	
